# Effect of Energy Input on Microstructure and Mechanical Properties of Titanium Aluminide Alloy Fabricated by the Additive Manufacturing Process of Electron Beam Melting

**DOI:** 10.3390/ma10020211

**Published:** 2017-02-21

**Authors:** Ashfaq Mohammad, Abdulrahman M. Alahmari, Muneer Khan Mohammed, Ravi Kottan Renganayagalu, Khaja Moiduddin

**Affiliations:** 1 Princess Fatima Alnijiris’s Research Chair for Advanced Manufacturing Technology (FARCAMT Chair), Advanced Manufacturing Institute, King Saud University, Riyadh 11421, Saudi Arabia; alahmari@ksu.edu.sa (A.M.A.); muneer0649@gmail.com (M.K.M.); kmoiduddin@gmail.com (K.M.); 2 Industrial Engineering Department, College of Engineering, King Saud University, Riyadh 11421, Saudi Arabia; 3 PSG Institute of Advanced Studies, Coimbatore 641004, India; krr@psgias.ac.in

**Keywords:** additive manufacturing, electron beam melting, titanium aluminide, computer tomography

## Abstract

Titanium aluminides qualify adequately for advanced aero-engine applications in place of conventional nickel based superalloys. The combination of high temperature properties and lower density gives an edge to the titanium aluminide alloys. Nevertheless, challenges remain on how to process these essentially intermetallic alloys in to an actual product. Electron Beam Melting (EBM), an Additive Manufacturing Method, can build complex shaped solid parts from a given feedstock powder, thus overcoming the shortcomings of the conventional processing techniques such as machining and forging. The amount of energy supplied by the electron beam has considerable influence on the final build quality in the EBM process. Energy input is decided by the beam voltage, beam scan speed, beam current, and track offset distance. In the current work, beam current and track offset were varied to reflect three levels of energy input. Microstructural and mechanical properties were evaluated for these samples. The microstructure gradually coarsened from top to bottom along the build direction. Whereas higher energy favored lath microstructure, lower energy tended toward equiaxed grains. Computed tomography analysis revealed a greater amount of porosity in low energy samples. In addition, the lack of bonding defects led to premature failure in the tension test of low energy samples. Increase in energy to a medium level largely cancelled out the porosity, thereby increasing the strength. However, this trend did not continue with the high energy samples. Electron microscopy and X-ray diffraction investigations were carried out to understand this non-linear behavior of the strength in the three samples. Overall, the results of this work suggest that the input energy should be considered primarily whenever any new alloy system has to be processed through the EBM route.

## 1. Introduction

Titanium aluminides (TiAl) are candidate materials for many high-temperature aero-engine gas turbine components such as turbo chargers and low-pressure turbine blades [1]. Because these alloys have less density and good high-temperature strength, they are also actively being considered for use in automotive components such as engine valves [2]. While considerable progress has been made in the past decade with regard to understanding these alloys and how to improve their basic mechanical properties, issues persist on how to fabricate the alloys in to complex-shaped parts.

Klocke et al. rated TiAl as difficult-to-cut alloys [3]. Excessive generation of heat while cutting leads to faster tool wear. Cryo-assisted cutting and high-speed cutting are some of the specialized techniques suggested to overcome the machining difficulties. Limited ductility of TiAl alloys at room temperature hinders conventional forging too. This has been overcome by isothermal thermal forging where the die is maintained at significantly higher temperatures. At present, compared to the two mentioned techniques, investment casting is opined to be a more suitable route for bulk manufacturing of TiAl components [4]. Even here, the high reactivity of TiAl with the melting crucible turns out to be one of the many technical problems yet to be overcome, apart from cost-benefit concerns. 

In this backdrop, metal based additive manufacturing stands out as a viable approach for TiAl alloys. In additive manufacturing, no cutting tools are used nor are there any crucibles. Complex shaped parts can be produced from the feedstock powder itself. The powder is spread in layers; each layer is bonded to the previous one by melting with a highly focused beam. Electron Beam Melting (EBM) is one such additive manufacturing technique that utilizes an electron beam to melt powders. The build chamber in EBM is under high vacuum, unlike its contemporary the Selective Laser Melting (SLM) process. The vacuum not only protects reactive alloys such as TiAl from oxidation, but the overall build temperature can be maintained well over the room temperature restricting the residual stresses in the final part.

Within a few years, EBM has made rapid strides in the bio-implant and aerospace manufacturing sectors. EBM proved to be a commercial success especially for Ti-6Al-4V alloy, which is a popular material for both of these sectors [5]. In this scenario, there is an opportunity to extend the EBM process to materials such as TiAl that are difficult to process by conventional methods. A few attempts have already been made in this direction. One of the earliest works on EBM of TiAl was reported by Cormeir et al. using both pre-alloyed and blended powders [6]. While EBM processing of TiAl was demonstrated to be feasible, at the same time loss of aluminum during EBM was identified as a problem. In another study, Murr et al. were able to produce TiAl samples with nearly 98% theoretical density. The EBM produced parts containing some residual porosity due to non-optimized build parameters as well as some Argon bubbles that were pre-existing in the starting powders themselves [7]. Franzén and Karlsson reported that although EBM can produce fully dense parts of TiAl in principle, the parts may not have the most desirable microstructure [8]. Hence, it can be summarized that most of the above problems could be overcome if the process parameters are carefully optimized. 

A number of parametric optimization studies are available in EBM, although they mostly pertain to Ti-6Al-4V. A few researchers studied individual EBM parameters and inferred that beam current and beam scan speed are important. These parameters directly influenced defects [9], microstructure [10], and mechanical properties [5]. Juechter et al. blended two parameters (beam power and scan speed) into a higher order framework called ‘line energy’ and showed that a minimum of line energy is required to achieve fully dense parts [11]. Line energy affected the amount of aluminum loss too. We undertook the present work on similar lines as we felt that such studies are needed if newer alloys such as TiAl were to ever occupy a predominant place within the library of EBM materials.

While it can be agreed that line energy is important as suggested by Juechter et al. [11], the effect of beam offset (the distance between two successive scan lines) was conspicuously absent within the line energy definition. In fact, Gong et al. showed that beam offset can significantly influence the porosity levels in an EBM part [12]. Hence, there is a need for a better energy index in EBM process optimization, wherein the combined effects of line offset, beam current, and scan speed should be accounted for. This was done in the current study by considering ‘area energy’. Previously, a similar attempt was made by Koerner et al. to see the effect of hatch strategy while keeping the area energy constant [13]. We produced TiAl samples using EBM at three levels of area energy inputs and the samples were analyzed for porosity, microstructure, and mechanical strength.

## 2. Materials and Methods

**Figure 1:**
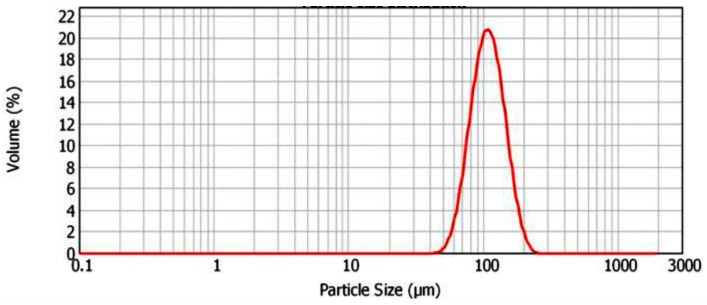
Powder particle size distribution measured by laser diffraction technique.

**Figure 2:**
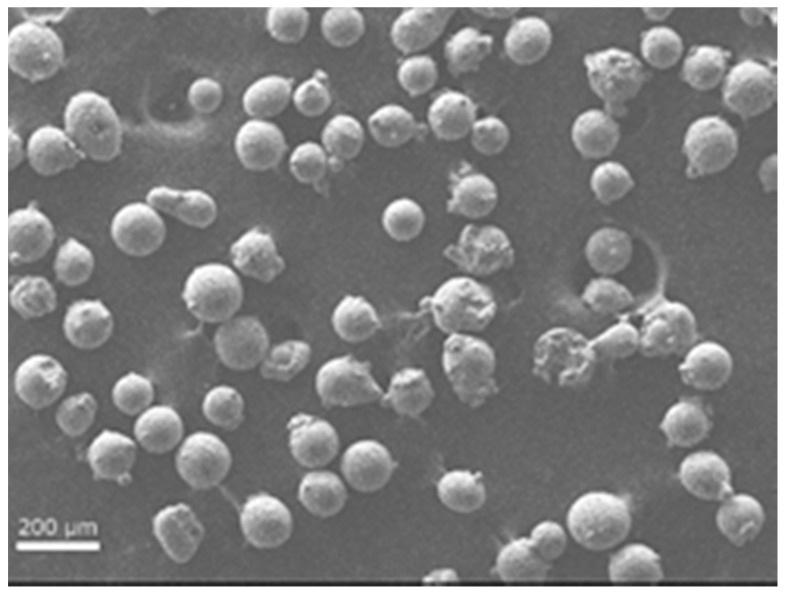
Scanning electron microscope image showing spherical powder particles.

The feedstock powder used in the current study was γ-TiAl (Ti-48Al-2Cr-2Nb), which was supplied by Arcam AB (Mölndal, Sweden). The chemical composition of the powder is given in Table 1. The powder particles were found to be in the size range 45–180 µm with a mean of around 110 µm (Figure 1). Particle morphology was more or less spherical, with smaller satellite particles adhering to the bigger ones (Figure 2).

**Figure 3:**
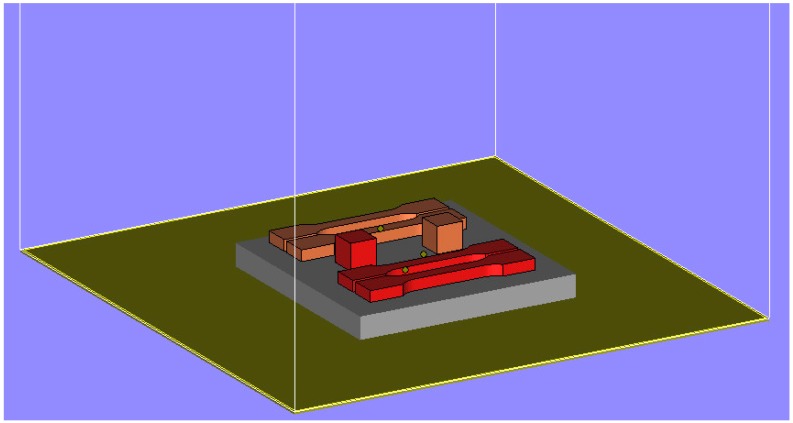
Arrangement of tensile and cube samples as modeled in Magics (Ver.17) software (Materialise NV, Leuven, Belgium).

EBM experiments were done on anA2 (Arcam AB) machine. More details about the EBM process can be found elsewhere [14]. Cubes having 12 mm sides were built along with tensile specimens (32 mm gauge length and 4 mm thick) (Figure 3). A square (100 × 100 mm) stainless steel plate of 10 mm thickness was used as the substrate upon which the parts were built. CAD representations of the samples were first saved in STL format in Magics (Ver.17) software (Materialise NV, Leuven, Belgium) and then transferred to the Build Assembler software (Arcam AB). This proprietary software from Arcam AB converts the STL geometry in to thin slices and writes the output as a machine-specific ABF file. The ABF file instructs the machine control how to proceed with the actual part production.

According to Kirchner et al. [15], line energy in the EBM process can be calculated as:

*E_l_* = *U_e_* × *I_b_*/*v_sc_* (1)

where *U_e_* is acceleration voltage, *I_b_* is beam current, and *v_sc_* is scan speed.

As noted earlier, line energy is not a complete measure of the amount of energy input during EBM. Since a number of overlapping scan lines constitute a given layer area, the amount of energy supplied to the layer per unit area can be calculated as:

*E_A_* = *E_l_*/*L_o_* (2)

where *E_A_* is Energy per unit area; and *L_o_* the line offset.

In the current study, three levels of area energy inputs were considered. Keeping the acceleration voltage (*U_e_*—60 kV) and beam scan speed (*V_sc_*—2200 mm/s) as constant, beam current and line offset values were varied to influence the area energy effectively as given in Table 2. First, we carried out trial runs to know the extremes of these two parameters. Beyond these extremes, the process became unstable. In this way, we found that the beam current must be between 15 A and 25 A and line offset between 0.17 mm and 0.35 mm. Once we fixed these outer ranges, we assigned the intermediate values, which were not the mathematical mean values. The intermediate values in Table 2 were chosen somewhat closer to the mean randomly. This was to avoid any systematic errors existing in the experimental space. This also allows capturing of non-linearity within a process. The powder bed was maintained at a temperature of 1100 °C throughout the fabrication process. For all the builds, the scan orientation was alternated by 90° after each layer.

Microstructures of the samples in as-built condition were examined using polarized light optical microscopy (Carl Zeiss Axio Scope A1; Zeiss, Oberkochen, Germay). Pore sizes distribution was analyzed with micro-CT (Phoenix VTomeX L240; General Electric, Fairfield, CT, USA). Phase analysis was carried out using X-ray diffraction (XRD) on Bruker’s Discover D8 (Bruker, Billerica, MA, USA). Vickers micro hardness tests were carried out on a Buehler Micromet 5144 machine (Buehler, ITW, Lake Bluff, IL, USA) using a load of 500 g for 10 s duration. Tensile testing as per ASTM E8 standard was done at a cross-head speed of 0.1 mm/s. Fractured tensile specimens were viewed under Scanning Electron Microscopy (SEM) (JEOL JSM-6610LV, Akishima, Japan).

## 3. Results and Discussion

### 3.1. Microstructure

Typical Ti-48Al composition is believed to have the best ductility among the titanium aluminide alloys [16]. The ductility is further improved by small additions of Cr, whereas Nb decreases oxidation attack at higher temperatures. Eventually, this kind of chemical composition leads to two phase microstructure. The two phases are α2(Ti3Al) and γ(TiAl). Four different varieties of microstructures could emerge depending upon the chemical and processing route: near γ, duplex, near lamellar, and fully lamellar [17].

**Figure 4:**
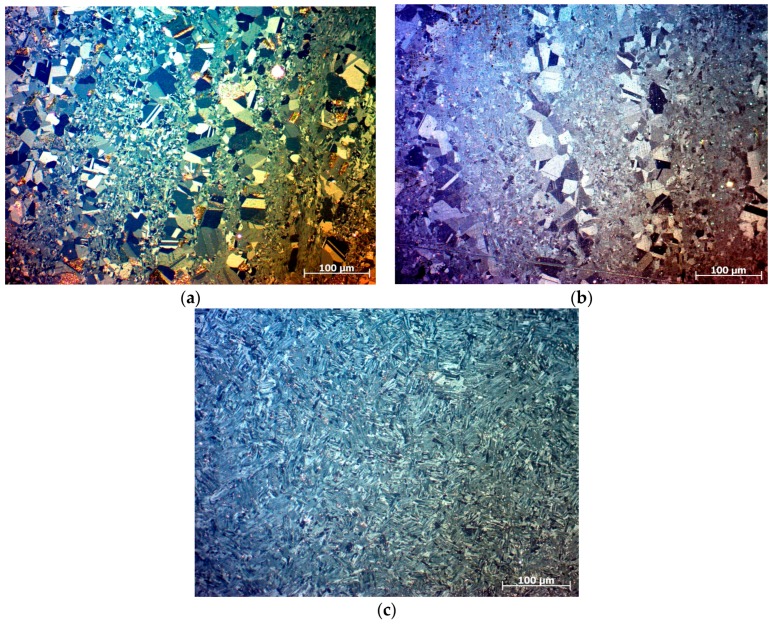
Cross-sectional microstuctures of γ-TiAl samples produced using of low energy (**a**); medium energy (**b**); and high energy (**c**) inputs. The build orientation is from bottom to top.

The microstructures of EBM samples corresponding to mid-sections (in both directions: along the height and thickness) are shown in Figure 4. When the energy was lowest, duplex microstructure was seen. The duplex microstructure consisted of equiaxed (γ) and lamellar (γ + α2) regions. When the input energy surged, the lamellar region started to grow at the expense of the equiaxed region; until there was almost nothing left of the equiaxed regions in the high-energy sample.

The microstructures observed here are from the mid-section of the samples. Therefore, it is reasonable to believe that the heat-cool cycles, produced by melting of the subsequent layers, have affected the initial microstructure. If the microstructure at the beginning of the heating cycle is duplex then γ → α transformation occurs; instead if it was already a lamellar microstructure, then thickening of the α phase occurs. Whatever the case, heating of the solidified phases to higher temperatures results in a greater amount of lamellar structure, this is what is seen in Figure 4, wherein a complete lamellar microstructure can be seen in the high-energy condition.

γ-TiAl powders with composition similar to our study were consolidated using a rapid consolidation process called Plasma Pressure Compaction [18]. Similar results to our study were reported; that is, higher consolidation temperature resulted in a fully lamellar microstructure, whereas lower temperature produced a duplex structure. Chandley too reported an equiaxed microstructure when the cast temperature was especially low for TiAl [2].

Bands of large grains were seen in the low and medium energy samples, whereas the same bands were absent in the high energy sample (Figure 4). In EBM, because each layer is melted on top of a previously solidified layer, the grains in the preceding layer assist in epitaxial growth in the subsequent layer [19]. Columnar grains were observed even in Ti-6Al-4V EBM samples [20]. Typically, in a melt pool, columnar grains grow parallel to the heat extraction direction, which in the present case is towards the bottom. The columnar buildup can be blocked and the grains can be refined if the temperature gradient at the solidifying front is below a critical value [21]. High energy input ushered such a shallow temperature gradient thus eliminating the columnar structure in Figure 4c.

**Figure 5:**
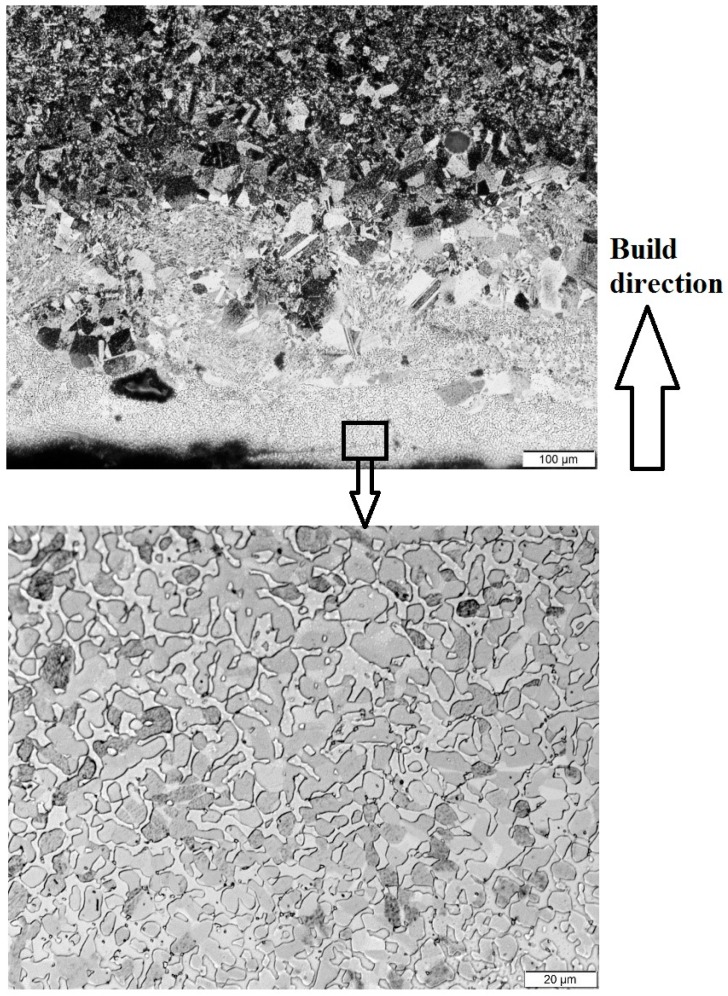
Microstructures from the bottom portion of the electron beam melting (EBM) part built on a steel support plate.

**Figure 6:**
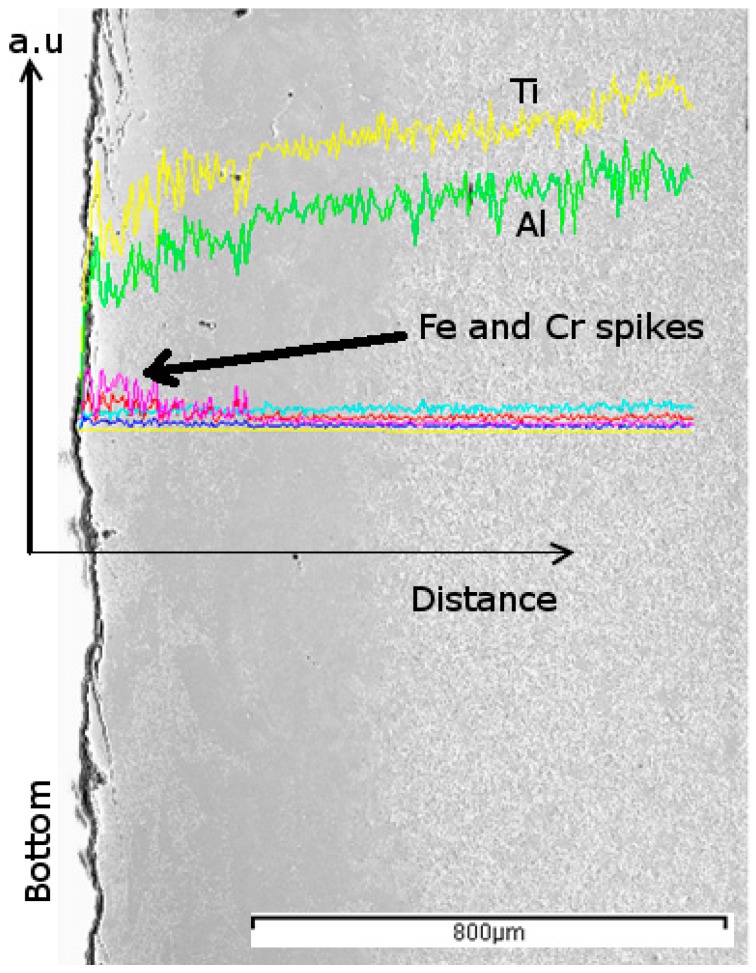
Scanning electron microscopy–Energy dispersive X-ray spectroscopy (SEM–EDS) linescan showing diffusion of elements from the substrate plate in to the built part (Yellow—Ti, Green—Al, Pink—Fe, Red—Cr, Cyan—Nb, Blue—Ni).

At the bottom of the high-energy sample, which is substrate/part interface, the microstructural features were distinct for a thickness of nearly 400 µm, as can be seen in Figure 5, which presumably contained many Fe-Ti intermetallics. Scanning electron microscopy–Energy dispersive X-ray spectroscopy (SEM-EDS) analysis across the base/build interface confirmed a considerable amount of iron in this region, Figure 6. This is to be expected: the substrate would melt at least when the first few layers are deposited, more so when the beam delivers high energy. When employing stainless steel base plates for building γ-TiAl parts, it is therefore necessary to completely machine off the diluted base/build interface region from the part proper. It has to be noted here that a certain amount of alloy mismatch is necessary between the substrate and the part as it was easy to dislodge the part after the build was complete.

**Figure 7:**
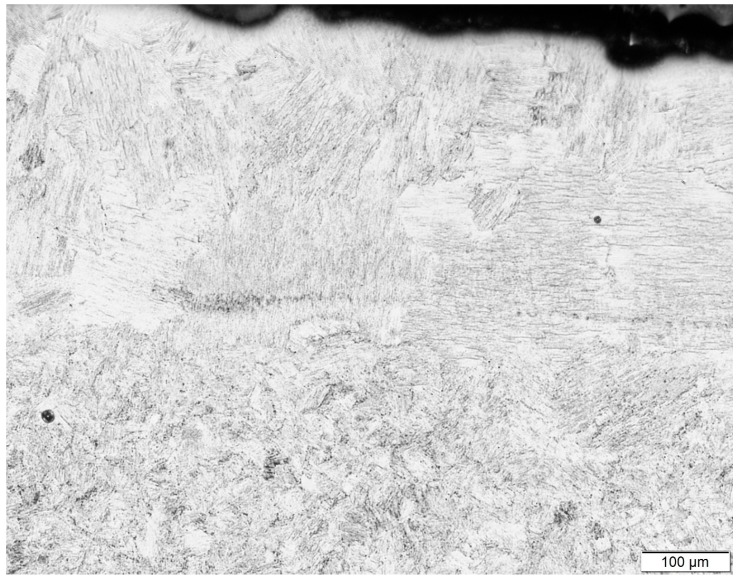
Coarse microstructure in the last layer(s) of the high energy sample.

The top most layer showed a slightly different microstructure compared to the layers underneath, as can be seen in Figure 7. The microstructure of the top most layer had coarse lamellar grains, whereas the layers below had a finer recrystallized structure. This confirms that every subsequently added layer has reheated and tempered the lamellar structure of the layer below [22]. Since the top layer was deposited last, it did not see any such post-heating effects.

The results of SEM–EDS analysis done at the center of the samples are given in Table 3. Considerable loss (close to 3 at%) in Al (due to vaporization) was observed in samples produced using high energy input. Schwerdtfeger and Körner also reported a similar loss during EBM processing of γ-TiAl at high energy inputs [23]. The loss of Al can account further for the fully lamellar microstructure observed in the samples produced using high energy input (Figure 4c).

**Figure 8:**
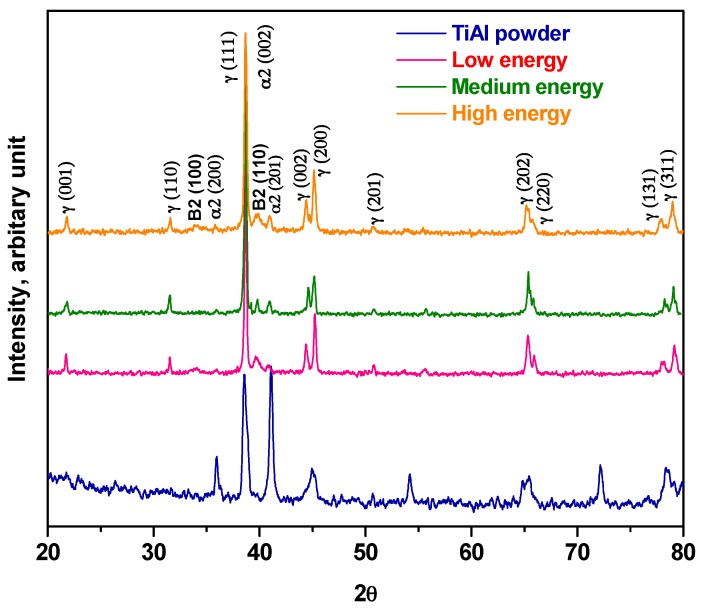
X-ray diffractograms of γ-TiAl samples produced using different energy inputs and feedstock powder.

Figure 8 compares the XRD peak profile of as-received powders with EBM-built TiAl samples at different energy inputs. The presence of both γ(TiAl) and α2(Ti3Al) phases can be seen in the case of as-received powders. Based on spectral area of the peak, the ratio of γ/α2 is found to be in the range of 0.4–0.5 for as-received powders. Similarly, a γ/α2 ratio indicating high amount of α2 in the feedstock powder was also reported by Murr et al. [7]. In contrast, the low energy sample showed the presence of γ-TiAl predominantly with a very small fraction of α2-Ti3Al phase. Similar observations were made for even the medium and high energy specimens. The γ/α2 ratio was found to be in the range of 0.9–0.95. This ratio is on the higher side as compared with earlier reports [6,24]. According to the equilibrium diagram the ratio of γ/α2 is controlled by the Al content. It can also be said that the cooling rate and heat treatment additionally determine γ/α2 ratio. Indeed, these two factors are inherent to an EBM process. When compared with as-received powders, the EBM-built TiAl samples showed the presence of additional β (B2) phase.

**Figure 9:**
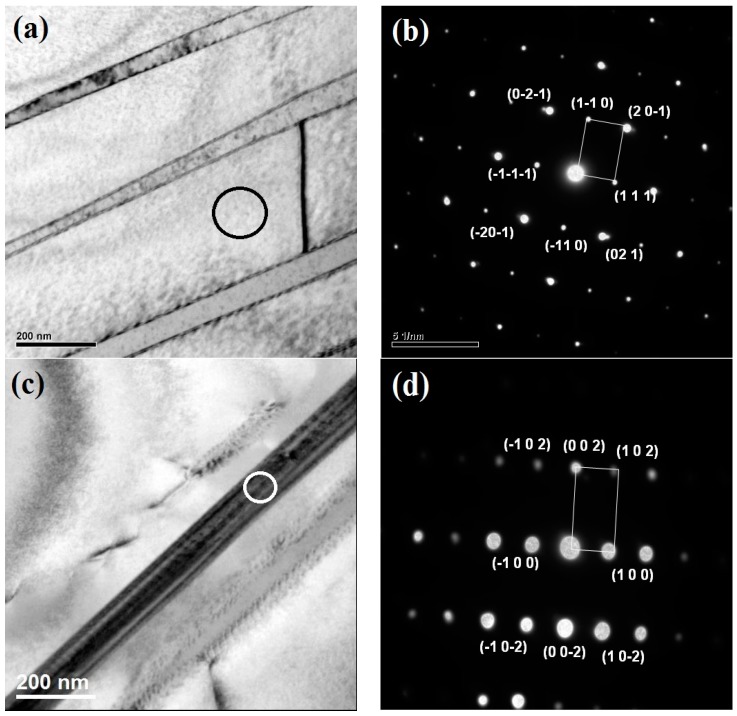

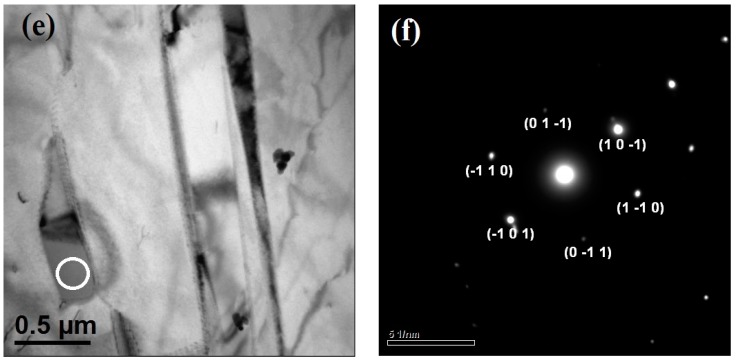
Bright field transmission electron microscopy (TEM) image of (**a**) γ (**c**) α2 and (**e**) β phase and the corresponding selected area electron diffraction( SAED) pattern taken from (**b**) [−1–1 2] (**d**) [0–1 0] and (**f**) [1 1 1] zone axis, respectively.

**Figure 10:**
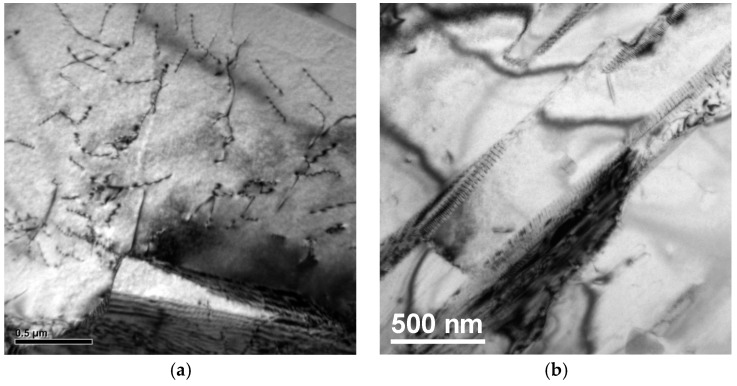
Bright field TEM image of high energy input EBM-built TiAl samples showing the dislocations (**a**) and twin structure (**b**).

Figure 9 shows the bright field transmission electron microscopy (TEM) images and corresponding selected area electron diffraction (SAED) pattern of EBM-built TiAl samples with high energy input. Presence of α2/γ laths can be observed in Figure 9a,c. The corresponding selected area diffraction (SAD) pattern taken from [−1–12] and [0–10] zone axis confirms the presence of γ (Figure 9b) and α2 (Figure 9d) phase, respectively. The high cooling rate associated with the EBM process also influences the lamellar spacing in the colonies and is measured to be an average of 0.4 µm for high energy input samples. The bright field TEM image in Figure 9e and the corresponding SAD pattern taken from [111] zone axis (Figure 9f) substantiates the XRD analysis on the B2 β-phase formation. According to the binary phase diagram of Ti–Al, B2 β-phase does not exist as an equilibrium phase. On the other hand, β is an equilibrium phase in the quaternary Ti–Al–Cr–Nb phase diagram at certain thermodynamic conditions. It exists over a wide concentration range, including the composition of the alloy studied Ti-48Al-2Cr-2Nb, and forms a variety of two-phase and three-phase phase equilibria involving α, γ, and α2 phases depending on the temperature. Instead of solidifying mainly through the α-phase the solidification pathway is switched to the Ti-rich side of the peritectic reaction and significant amounts of β-phase are formed on solidification from the melt during faster cooling associated with the EBM process. The subsequent “heat treatments” are also affected as the temperature range spent in the α-single phase field is extended by the loss of Al, and in the extreme case of very high Al losses, even the pure β-phase field might be reached. Figure 10 displays dislocations and twins in γ grains. These defects emerge mainly because of the rapid solidification rates experienced in EBM. A high dislocation density in the γ-phase is a primary reason for the high hardness and strength of EBM γ-TiAl [7]. In addition to that, the formation of a fine lamellar structure with smaller inter-lamellar spacing can play an important role in the enhancement in strength of EBM built TiAl samples.

### 3.2. Micro-CT

**Figure 11:**
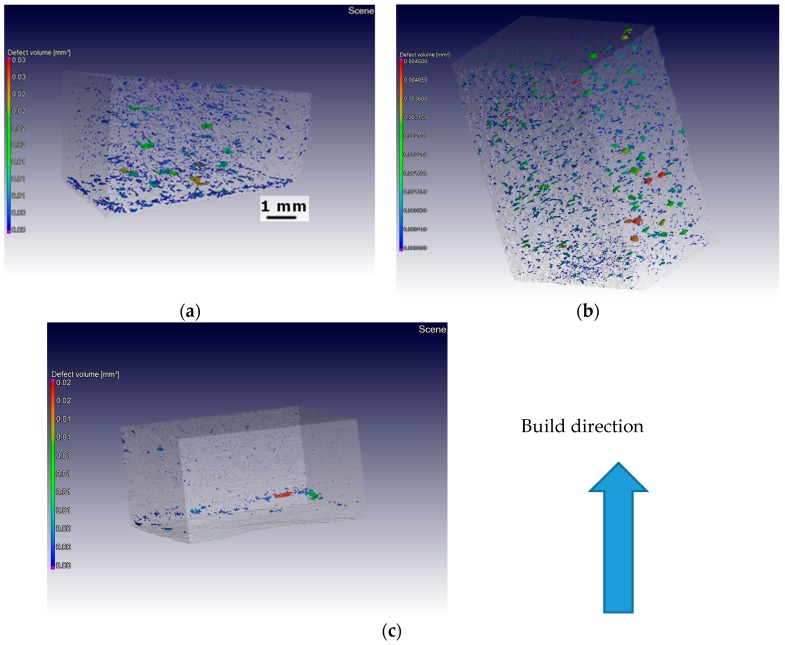
Micro-computed tomography (micro-CT) representation of pores/imperfections in γ-TiAl samples produced using low (**a**); medium (**b**); and high (**c**) energy input samples.

Samples were analyzed using micro-computed tomography (micro-CT) and the results are given in Table 4 and Figure 11. In samples produced with low energy input, the pores were bigger in dimensions and larger in number, and were distributed more or less uniformly over the entire volume. On the other hand, in the samples produced using high energy input, the number of pores decreased drastically. The pores were confined to the bottom region. This was expected, as it takes several layers before the powder starts to spread and melt uniformly.

The bigger sized defects as seen in Figure 11 mostly lie along the layers, i.e., perpendicular to the build direction. These defects typically occur in between layers and they were more prevalent in the samples produced using low energy input, suggesting improper fusion of the layers.

In the medium energy sample, the porosity levels were in between low and high energy conditions. Interestingly, the largest pore in the medium energy condition was almost 50% smaller in diameter than in the other two cases (Table 4). The same is confirmed by Figure 11. In fact, the defect volume scale is less by an order of magnitude in the case of the medium energy sample. Therefore, it can be attested that medium energy input was better in terms of pore sizes.

### 3.3. Mechanical Properties

**Figure 12:**
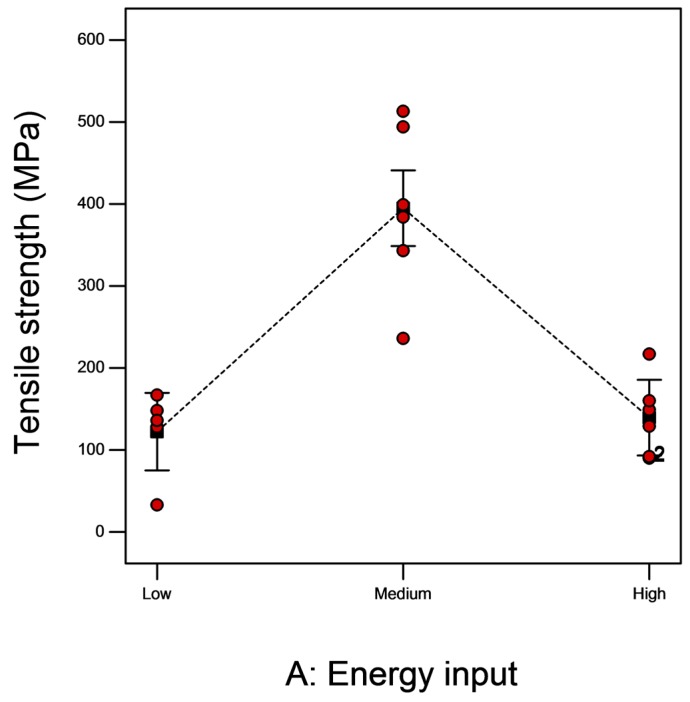
Effect of energy input on tensile strength.

The ultimate tensile strength values measured on specimens are given in Figure 12. Samples produced using medium energy showed considerably higher tensile strength. The strength values of these samples are close to wrought form (480 MPa) [25] but superior to castings (275–380 MPa) [26]. The ultimate tensile strength of the medium samples is at par with those reported for EBM γ-TiAl in earlier investigations [8,24]. The presence of a two phase microstructure ultimately resulted in the best combination of properties.

**Figure 13:**
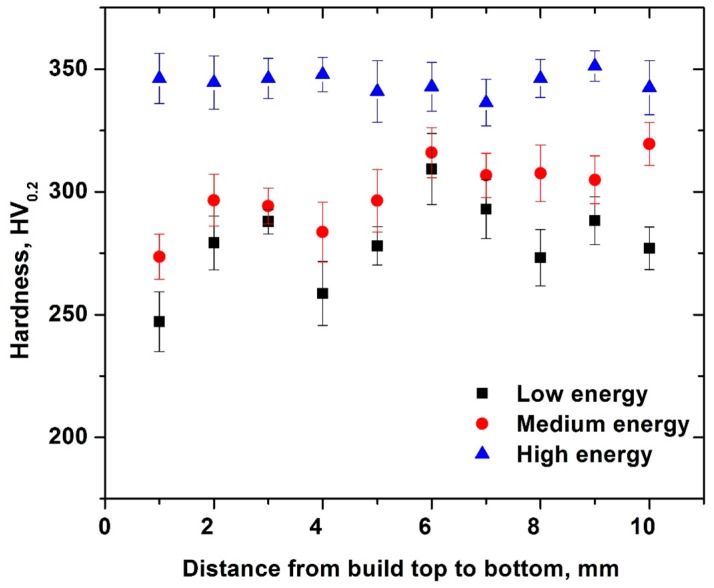
Microhardness distribution from the top to bottom portion of cubes.

The results of microhardness measurements along the sample height are shown in Figure 13. As expected, samples produced using a high energy input showed consistently higher hardness than those produced using a low energy input.

**Figure 14:**
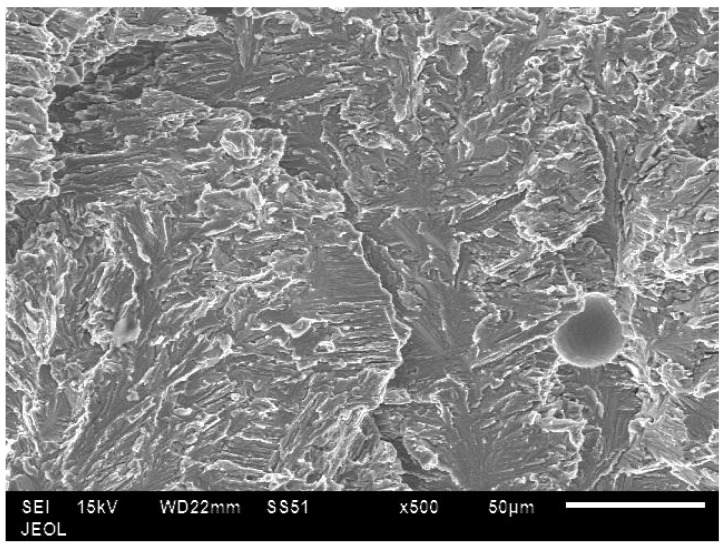
SEM fractograph showing cleavage type of fracture.

**Figure 15:**
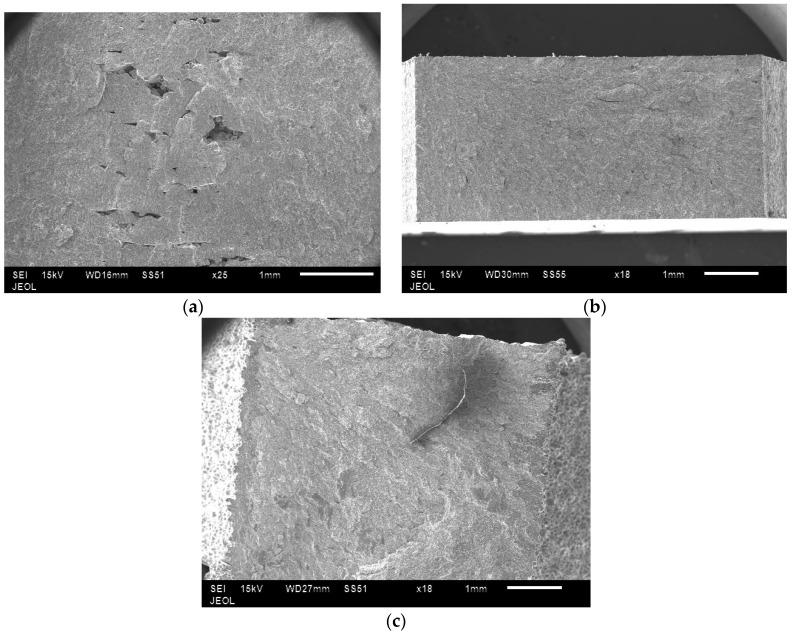
Lack of bonding between layers seen on the tensile fracture surfaces of low energy samples (**a**). These defects were absent in the samples produced using medium (**b**) and high (**c**) energy input.

**Figure 16:**
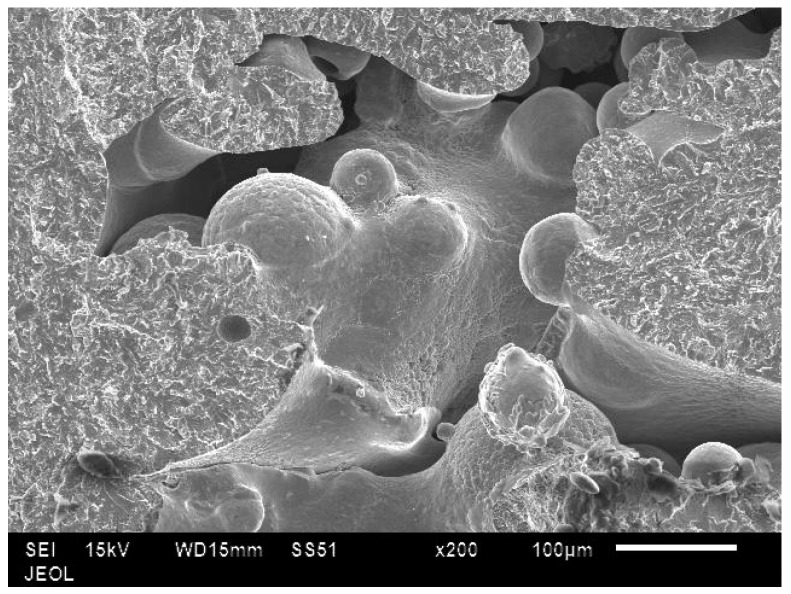
Unmelted powder particles seen on the tensile fracture surfaces of low energy samples.

In all the samples, tensile fractures were found to occur predominantly in cleavage mode (Figure 14). In low energy samples, lack of bonding between layers could be clearly seen (Figure 15). In these samples, unmelted powder defects were also noticed (Figure 16). These observations confirm why low energy samples showed inferior tensile strength compared to high energy samples.

**Figure 17:**
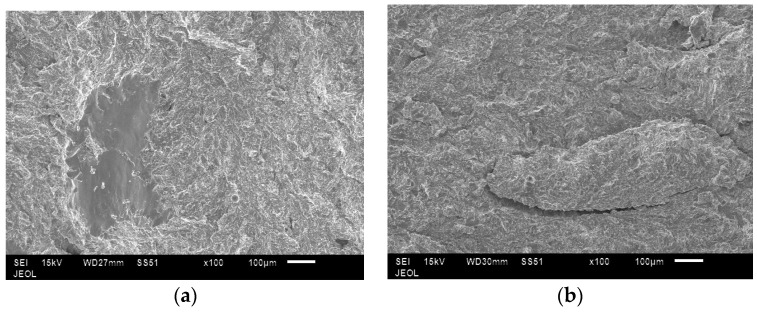
Presence of keyhole defect in high energy sample (**a**) Evidence of shear lip features in medium energy (**b**) Fracture surfaces.

Few flat zones with no trace of any fracture features were seen in the high energy fracture surface (Figure 17). µ-CT analysis indicated that the number of pores in the high energy samples were notably less than in the other two samples. Nevertheless, the pores present were bigger in size when compared to the medium energy input. The beam with high current input and packed scan spacing could have caused key hole defects. This was similar to defects seen in the selective laser melting process [27]. A highly intense beam produces a deep melt pool with a wide upper head and narrow bottom. Gas can easily get trapped in the confined bottom portion of the melt pool without any escape route. Another complication in EBM is that the highly energetic beam can ‘blow’ the powder away in some regions. This problem was felt more acutely when the current was increased and the scan spacing reduced, both at the same time. These defects finally affected the overall tensile strength of the high energy samples. In general, components with micropores are highly susceptible to fatigue failure as these pores act as stress-raisers during service. Commonly, hot isostatic pressing (HIPing) can shrink down pores with reports suggesting even worst-case EBM samples have improved in quality by deploying this post-processing technique [28]. 

**Figure 18:**
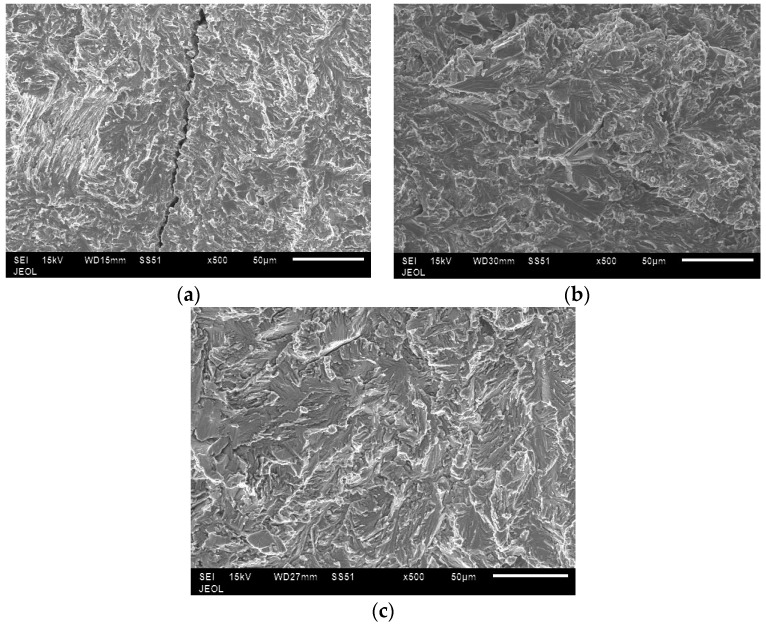
Fractographs of broken specimens in the tensile test; microcracks seen in the case of low energy (**a**); a mixed mode fracture in the medium energy condition (**b**); and lamellar delamination seen in the high energy condition (**c**).

In medium energy samples, there was evidence of shear lips (Figure 17). In lower energy input, the fracture was predominantly intergranular cleavage type (Figure 18). A micro-crack was also seen along the grain boundaries in the same sample. As the energy input increased, the fracture gradually turned to a transgranular cleavage type. It was proposed that internal stresses in the α2 phase are in tension and in the γ phase they are in compression [29]. This incompatibility in internal stresses causes delamination along the phase boundaries when the volume fraction of the lamellar grains dominates. This could be another reason for the low strength exhibited by the high energy sample besides the key-hole defects.

## 4. Conclusions

The effect of energy input was studied in EBM processing of γ-TiAl. The energy input was varied by changing the beam current and the line offset. The following conclusions can be drawn from the current study:

- In EBM, scan distance is one among other main parameters that decides consolidation of each powder layer. Because the scan distance parameter was absent in the line energy index, we used a higher order term called ‘area energy’ as a criterion to determine the effects on the build quality.
- Only the right amount of energy results in a duplex microstructure. Lower energies produce a predominantly equiaxed microstructure and relatively higher energies result in a completely lamellar microstructure.
- Lower energies resulted in lack of bonding between adjacent layers thus leading to high-aspect defects lying along the direction of the layers.
- The highest amount of energy reduced the percentage volume of the pores; however, the same intense beam also produced key-hole defects. The near-complete lamellar microstructure combined with such large sized defects rendered the high-energy samples weaker. Whereas, the right amount of energy, seen in the case of medium energy samples, produced a better microstructure as well as negligibly smaller sized pores, thereby resulting in the strongest components.

## Figures and Tables

**Table 1:** Chemical composition of feedstock powder.

Element	Al	Cr	Nb	Fe	C	O	N	H	Ti
Weight %	33.9	2.3	4.6	0.04	0.01	0.09	0.01	0.002	Bal.

**Table 2:** Process parameters used for fabrication of samples.

Parameter	Low Energy	Medium Energy	High Energy
Beam current, *I_b_* (mA)	15	19	25
Line offset, *L_o_* (mm)	0.35	0.20	0.17
Line energy, *E_l_* (J/mm)	0.34	0.54	0.69
Energy per unit area, *E_A_* (J/mm^2^)	0.97	2.71	4.07

**Table 3:** Scanning electron microscopy–Energy dispersive X-ray spectroscopy (SEM–EDS) analysis at the center of γ-TiAl samples produced using low and high-energy inputs.

Element	Low Energy Input (at%)	Medium Energy Input (at%)	High Energy Input (at%)
Al K	44.35	44.40	41.30
Ti K	51.80	52.20	54.70
Cr K	1.93	1.99	2.01
Nb L	1.92	1.91	1.92

**Table 4:** Porosity measurements using micro-computed tomography (micro-CT) (Volume studied: ~250 mm^3^).

Sample	Average Porosity (vol%)	Largest Pore Diameter (mm)	Total Number of Defects Detected
Low energy	0.26	1.53	4493
Medium energy	0.16	0.47	3807
High energy	0.03	1.03	560
